# Effects of low-intensity blood flow restriction training on myocardial injury indices, antioxidant and anti-apoptotic capacity in rats

**DOI:** 10.3389/fphys.2025.1508305

**Published:** 2025-03-17

**Authors:** Yuwen ShangGuan, Kunyi Huang, Zining Zhu, Yuan Yuan, Yawei Song, Hao Wang, Liang Chen, Shiqi Yu, Guangzhi Zheng, Qi Liang

**Affiliations:** ^1^ Institute of Competitive Sports, Nanjing Sport Institute, Nanjing, China; ^2^ Department of Exercise Physiology, Kunsan National University, Gunsan, Republic of Korea; ^3^ Department of Health and Physical Education, The Education University of Hong Kong, Tai Po, Hong Kong SAR, China; ^4^ School of Sports and Health, Shanghai University of Sport, Shanghai, China; ^5^ School of Sports and Health, Linyi Vocational College, Liny, China

**Keywords:** blood flow restriction, low-intensity pressurized resistance training, myocardial injury, apoptosis, rats

## Abstract

**Objective:**

This study aims to investigate the effects of low-intensity blood flow restriction training on myocardial tissue in rats. By measuring the levels of myocardial injury biomarkers in serum and the expression of anti-apoptotic and antioxidant proteins in myocardial tissue, the study preliminarily explores the underlying mechanisms.

**Methods:**

Male 3-month-old Sprague-Dawley rats were randomly divided into the following groups: control group (CON), low-intensity training group (LIRT), high-intensity training group (HIRT), and low-intensity blood flow restriction training group (LIBFR), with 6 rats in each group. Body weight, maximum voluntary carrying capacity, myocardial morphology, myocardial injury biomarkers, and the expression levels of Bcl-2, Bax, Nrf2, and Keap1 proteins in myocardial tissue were evaluated.

**Results:**

(1)cTn1 Detection: The HIRT group showed a significant increase in cTn1 levels (P < 0.01), while the LIBFR group had a lower cTn1 level compared to the HIRT group (P < 0.05). (2)Nrf2 and Keap1 Results: Compared to the CON group, the LIBFR group showed an increase in Nrf2 (P < 0.05), and a significant increase in Keap1 (P < 0.01). (3)Bcl-2 and Bax Results: Compared to the CON group, Bcl-2 levels were significantly elevated in the HIRT group (P < 0.01) and increased in the LIBFR group (P < 0.05), while Bax expression was significantly reduced in the LIBFR group (P < 0.05). Regarding the Bcl-2/Bax ratio, the LIRT, HIRT, and LIBFR groups exhibited significantly higher values compared to the CON group (P < 0.01). Furthermore, the HIRT and LIBFR groups showed significantly higher Bcl-2/Bax ratios than the LIRT group (P < 0.01).

**Conclusion:**

Low-intensity blood flow restriction training can effectively reduce cTn1 in rat serum, decrease cardiomyocyte apoptosis, and improve antioxidant capacity, which has a certain protective effect on the myocardium.

## Introduction

Exercise is a unique form of stress that causes both external and internal stress on the body, impacting various physiological systems. Excessive exercise can cause physical damage, especially myocardial injury (Lavie et al., 2015). Under normal circumstances, the myocardium has a certain degree of self-protective function. Although exercise may cause reversible myocardial ischemia and hypoxia in the myocardium, it does not cause damage to the heart, which may be related to the defense system of the heart itself (Cornet et al., 2013). Regular moderate-to-vigorous intensity aerobic exercise (such as brisk walking, jogging, cycling) has a positive impact on cardiometabolic health, and can improve cardiopulmonary exercise capacity, blood pressure, blood sugar control, hypercholesterolemia and vascular endothelial function (Otsuki et al., 2016; Cavalcante et al., 2023). However, overly intense vigorous exercise may cause acute inflammatory responses in the myocardium, and myocardial inflammation is one of the important causes of cardiovascular diseases and sudden cardiac death in athletes (Compagnucci et al., 2021). High intensity resistance training (HIRT) is commonly used to improve muscle strength and vascular function (Lixandrão et al., 2018). However, high-intensity training may not be appropriate for individuals with cardiovascular risk factors such as hypertension, coronary heart disease, and obesity (Minniti et al., 2020). These populations often face significant challenges when participating in high-intensity exercise programs due to the potential stress on the cardiovascular system (Wewege et al., 2018). Therefore, we need a safe and effective method that improves muscle strength and promotes fitness in patients while reducing cardiac stress.

Low Intensity Blood Flow Restriction Training (LIBFR) is a newly emerging training method in recent years (Rolnick et al., 2021). This training method has been widely recognized as safe and effective (Centner et al., 2019; Mannozzi et al., 2023). LIBFR can significantly reduce the burden on the cardiovascular system through the use of low-load training modalities, while activating the body’s physiological response by way of blood flow restriction to achieve a similar effect to that of high-intensity resistance training (Schoenfeld et al., 2015). This makes LIBFR an ideal option, especially for groups at higher risk of cardiovascular disease or the elderly, who are often unable to withstand the burdens associated with high-intensity training. An increasing number of studies have shown that using low-load resistance training (<50% 1 R M) combined with blood flow restriction can effectively enhance muscle strength (Scott et al., 2015), and can even achieve the effect of high-intensity resistance training (HIRT) (Laurentino et al., 2012; Ozaki et al., 2013). Despite this, comparisons of effectiveness between LIBFR and HIRT remain controversial. Some studies have noted that LIBFR has higher muscle strength and hypertrophy than HIRT (Zhiyuan et al., 2019), while others have shown that HIRT is more effective than LIBFR (Vechin et al., 2015). Therefore, the applicability and efficacy of LIBFR in patients with cardiovascular disease and the elderly still require further research and validation. Given this need, we will adopt the method of combining blood flow restriction with low-intensity resistance training, which can be applied to individuals with cardiovascular diseases, geriatric conditions, and those unable to perform high-intensity exercises (Ogawa et al., 2021).

Regarding the study of LIBFR in animal models, the results of existing studies have shown that LIBFR exhibits significant antioxidant and muscle-enhancing effects in rat models (Pearson and Hussain, 2015; Bahreinipour et al., 2018; Garcia et al., 2022). However, there is still a lack of systematic research on the specific effects of LIBFR on the heart, especially in the context of cardiovascular disease. In some animal experiments, LIBFR has also shown potential to improve cardiac structure and function (Naderi-boldaji et al., 2018), but these studies have typically focused on adaptive responses in muscle and blood vessels, and studies related to myocardial injury remain scarce (Bejeshk et al., 2018). Therefore, in order to fill these research gaps, we explored the role of LIBFR in animal models and assessed the effects of LIBFR on the myocardium by indicators such as serum markers of myocardial injury and the expression of related proteins in myocardial tissues. We hypothesized that low-intensity blood flow restriction training (LIBFR) could attenuate myocardial injury in rats by modulating the expression of anti-apoptotic and antioxidant proteins and, to some extent, have similar or superior effects compared with high-intensity training (HIRT). We hope that this experiment will provide more information about the effects of the LIBFR training method on heart health, especially in cardiovascular risk groups, and thus provide a theoretical basis and experimental data for the future promotion of this method in clinical applications.

## Material and methods

### Animals

Twenty-four 3-month-old clean-grade male SD rats (approved by the Animal Ethics Committee of Nanjing Sport Institute), with a body weight of approximately 240g, were selected. They were housed in separate cages with the national standard rodent feed, 6 rats per cage, with free access to water and food. The temperature of the animal breeding room was 22°C ± 2°C, and the relative humidity was 30%–45%. After 1 week of adaptive breeding, they were weighed, numbered, and randomly grouped by computer-generated randomization into 4 groups: control group (CON), low-intensity training group (LIRT), high-intensity training group (HIRT), and low-intensity combined with blood flow restriction training group (LIBFR), with 6 rats in each group. A double-blind approach was adopted, where both the experimenters performing the interventions and the data analysts were blinded to group assignments to prevent bias.

### Sample size calculation

A power calculation was performed to determine the sample size for each group. Based on an expected medium effect size (Cohen’s d = 0.8) and a significance level of α = 0.05, a power of 80% was targeted. The sample size calculation indicated that 6 rats per group would provide sufficient statistical power to detect significant differences between groups.

### Training plan

**FIGURE 1:**
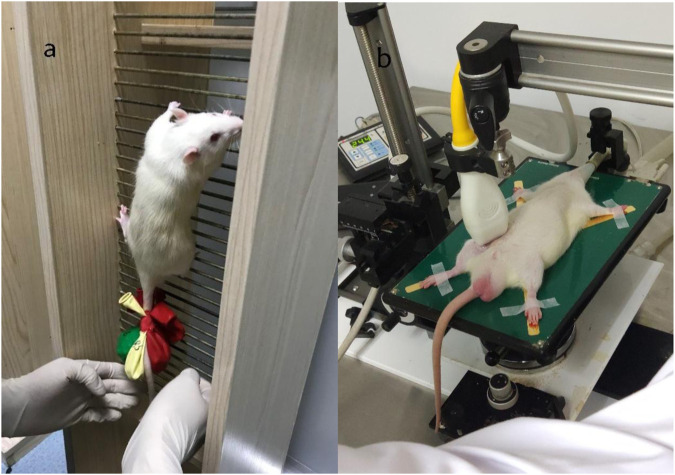
Rat weight-bearing ladder climbing training [left **(A)**], Color ultrasound detection of small animals [right **(B)**].

A 1-m-high climbing ladder was used, with an inclination angle of 85°, and there were a total of 54 steps, with each step spaced 0.5 cm apart. Above the steps was a platform that could accommodate up to 2 rats to rest simultaneously. The weight-bearing method was weight-bearing at the tail end of the rats (Song et al., 2023). As shown in Figure 1A, the rats were trained from Monday to Friday afternoon every week for 8 weeks. The specific exercise protocol is as follows:

CON: Normal breeding without any intervention.
LIRT: 30% MVCC loading was applied to the tail, climbing from the bottom to the top, 15 times, with an interval of 1 min between groups.
HIRT: 70% MVCC loading was applied to the tail, with 70% MVCC loading, climbing 15 times, with an interval of 1 min between groups.
LIBFR: 30% MVCC loading was applied to the tail and rubber bands were tied at the base of both thighs for 30% blood flow restriction. The rats climbed 15 times, with an interval of 1 min between groups. During the interval, the rubber bands were removed to allow normal blood flow.

### Blood flow restriction intervention method

**FIGURE 2:**
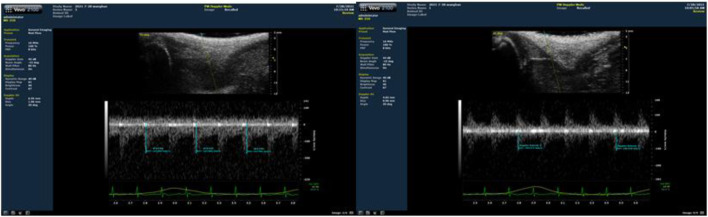
Ultrasound detection of the proportion of blood flow restriction (The left picture is before blood flow restriction, and the right picture is after restriction).

As shown in Figure 1B, blood flow velocity was detected at the root of the rat’s lower thigh using a small-animal-specific high-frequency color ultrasound (Visual Sonics Co., Toronto, Canada), and rubber bands of appropriate lengths were selected to encircle the thigh roots of the lower limbs of rats to ensure that the blood flow velocity was restricted to 30%–40% (Figure 2), Remember the length of the rubber bands used for each rat, which refers to the length of the restriction band that should be applied in the subsequent training sessions. During training, binding was performed at 1-min intervals, and blood flow restriction was lifted and blood flow reperfusion was performed (Tan et al., 2023). After rest, the area was bound again with rubber bands. In order to minimize the error of the rat’s lower limb size with growth changes and to ensure that the blood flow restriction was within the specified range, ultrasonic probing was performed every 3 weeks, and the length of the rubber bands was appropriately adjusted during training. In addition, the experimenters were trained to recognize signs of excessive blood flow restriction (e.g., unusual movement or skin discoloration) and to stop the intervention as soon as these signs appeared and make appropriate adjustments.

### Sample collection

One day before sampling, no intervention was carried out. Food was stopped at night, and an appropriate amount of water was provided. Before sampling, the weight of each rat was measured in sequence using an electronic balance and recorded according to the label. Anesthesia was performed using 10% chloral hydrate at a dose of 300 ug/100 g. Then, the anesthetized rats were placed in the supine position on an ice plate. The skin and subcutaneous tissues were cut in sequence, the abdominal aorta was separated for blood collection. The blood was left to stand at room temperature for 2 h and then centrifuged in a centrifuge (3,000 r/min, 15 min). After that, the upper serum was extracted using a rubber dropper, labeled, and stored in a −80°C refrigerator for future use. The mouse hearts were quickly removed and placed in pre-cooled 4°C physiological saline to clean the accumulated blood as much as possible. The surface moisture was absorbed with filter paper, and the weight was measured using an electronic scale. A part of the samples was fixed with paraformaldehyde and stored at 4°C for the preparation of tissue sections. Another part was placed in an EP tube, labeled, and stored in a −80°C refrigerator for future use.

### Hematoxylin-eosin staining

The heart tissues were fixed with paraformaldehyde for 48 h and then subjected to histological staining. This includes fixation, dehydration, clarification, embedding, sectioning, dewaxing, hematoxylin staining, eosin staining, re-dehydration, clarification and mounting.

### ELISA

Enzyme-linked immunosorbent assay (ELISA) was used to detect markers of myocardial injury and indicators of oxidative stress in rats. Serum samples were extracted and the concentrations of CK-MB, cTn1, SOD and MDA were measured using the corresponding ELISA kits (Wuhan Xavier Biotechnology Co. Ltd.). Standard curves are generated for each assay to ensure measurement accuracy. Positive and negative controls are included with each assay to confirm assay performance. All reagents and samples were handled according to the manufacturer’s instructions to ensure consistency in reagent volumes, incubation times and sample handling. The biochemical indicators in this study are explained below:

CK-MB: This enzyme is released into the bloodstream when myocardial injury occurs and is commonly used as a specific biomarker for detecting myocardial infarction or injury Elevated levels of CK-MB reflect cardiomyocyte damage and can be used to assess the extent of myocardial injury.
cTn1: Cardiac troponin I is a regulatory protein found in cardiomyocytes. It is highly sensitive and specific for myocardial injury. Elevated serum levels of cTn1 are indicative of myocardial cell damage and are often used as a diagnostic indicator of acute myocardial infarction.
SOD: Superoxide dismutase is an important antioxidant enzyme that protects cells from oxidative stress by neutralising superoxide radicals; elevated SOD activity is indicative of the body’s response to oxidative damage, which can occur in a wide variety of physiological and pathological processes, including cardiovascular disease and myocardial injury.
MDA: Malondialdehyde is a product of lipid peroxidation and is an indicator of oxidative damage. Higher levels of MDA reflect increased oxidative stress and cell membrane damage, which is often associated with conditions such as myocardial injury and myocardial ischaemia.

### Western blot

An appropriate amount of myocardial tissue was taken, RIPA lysis buffer was added, and the supernatant was obtained by centrifugation to obtain the protein sample. Then, electrophoresis was carried out to separate the proteins. The electrophoretically separated proteins were transferred from the gel to a PVDF membrane or nitrocellulose membrane for antibody incubation. After membrane washing, color development or luminescence detection was performed to obtain the protein band signal. The gray level was analyzed using the image analysis software Image Lab.

### Statistical analysis

After collating the data, graphs were plotted using GraphPad Prism8 and data were statistically analysed using SPSS 25.0 Normality of data was assessed using Shapiro-Wilk test (SW test). For normally distributed data, one-way analysis of variance (ANOVA) was used to compare means between groups. Non-parametric tests (Kruskal-Wallis test) were used for data that did not conform to normal distribution. Post hoc analyses were conducted using the LSD test for normally distributed data to further determine specific between-group differences. Additionally, Cohen’s d was calculated to assess the effect size for between-group differences using SPSS 25.0. p < 0.05 indicates significant differences, and p < 0.01 indicates a highly significant level.

## Result

### Body weight and MVCC

As shown in Table 1, the rat body weight and MVCC of the three trained groups all showed significant changes (P < 0.01). The MVCC of the HIRT group was higher than that of the LIRT group (P < 0.05), and there was no significant difference among the other groups (P > 0.05).

**TABLE 1:** Changes in rat body weight and MVCC.

Groups	CON	LIRT	HIRT	LIBFR
Initial body weight (g)	236.9 ± 5.8	234 ± 4.75	245.45 ± 4.2	230.97 ± 5.17
Final body weight (g)	607 ± 27.5	495 ± 24.7**	503.17 ± 30.06**	513.83 ± 27.48**
Cohen’s d	4.29	4.29	3.60	3.39
p-values	0.0000	0.0000	0.0000	0.0000
Initial MVCC(g)	280.67 ± 50.7	264 ± 44.33	257.83 ± 33.71	269.67 ± 40.53
Final MVCC(g)	652.16 ± 24.7	1,040.3 ± 85.3**	1,148.5 ± 53.24^#^**	1,103.83 ± 141.18**
Cohen’s d	5.00	6.10	5.50	0.90
p-values	0.0000	0.0000	0.0000	0.0000

Compared with the CON, group,**P < 0.01; Compared with the LIRT, group, ^#^P < 0.05.

Cohen’s d denotes effect size and p denotes test of statistical significance.

### HE staining

**FIGURE 3:**
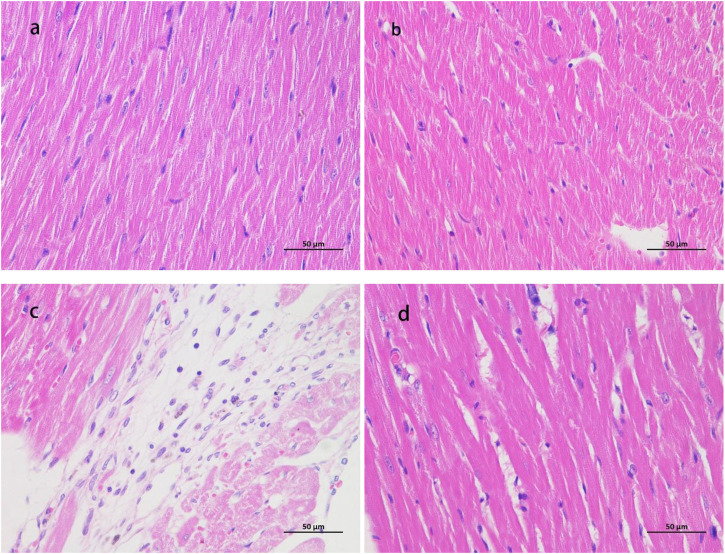
HE staining samples ×400 magnification **(A)** is the myocardial staining section of the CON group; **(B)** is the staining section of the LIRT group; **(C)** is the staining section of the HIRT group; **(D)** is the staining section of the LIBFR group) cTn1 and CK-MB.

As shown in Figure 3, the myocardial fibers in the CON group were normal. In the LIRT group, the myocardial fibers were evenly colored, had a consistent direction, the cell boundaries were clear, and no abnormalities were observed in the interstitium; no obvious inflammatory cell infiltration was seen. In the HIRT group, occasional proliferation of a small amount of connective tissue around the epicardium was observed in the myocardial tissue, accompanied by a small amount of lymphocyte infiltration and a small number of vacuoles. The arrangement, structure, and texture of the myocardial fibers were unclear and disordered, but no obvious abnormal changes were found in the cell nucleus and microvessels. In the LIBFR group, the myocardial fibers were evenly colored, had a consistent direction, the cell boundaries were clear, and no abnormalities were observed in the interstitium; no obvious inflammatory cell infiltration was seen.

### cTn1 and CK-MB

**FIGURE 4:**
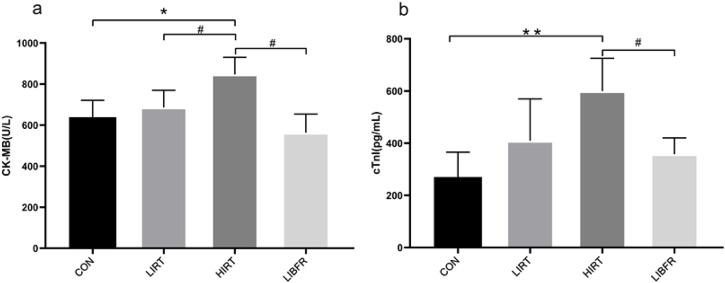
Concentrations of myocardial injury markers in rat serum of each group, **(A)** represents the determination result of CK-MB, **(B)** represents the determination result of cTn1.

As shown in Table 2; Figure 4. In terms of the CK-MB index, the serum CK-MB concentration value of the HIRT group was the highest, showing a significant difference compared with the LIRT group (P < 0.05), and a very significant difference compared with the LIBFR group (P < 0.01). In terms of the rat serum cTnI index, a significant difference was shown between the LIBFR group and the HIRT group (P < 0.05).

**TABLE 2:** Contents of myocardial injury markers in rat serum.

Groups	CON	LIRT	HIRT	LIBFR	Cohen’s d	p-value
CK-MB (U/L)	638.88 ± 82.42	686.14 ± 83.04^#^	847.32 ± 101.87*	562.9 ± 90.9^##^	1.69	0.019
cTnI (pg/mL)	270.48 ± 95.22	409.27 ± 160.48	599.85 ± 125.25**	358.13 ± 62.30^#^	1.27	0.044

Compared with the CON, group,*P < 0.05 Compared with the HIRT, group, ^#^P < 0.05, ^##^P < 0.01.

Cohen’s d denotes effect size and p denotes test of statistical significance.

### SOD and MDA

**FIGURE 5:**
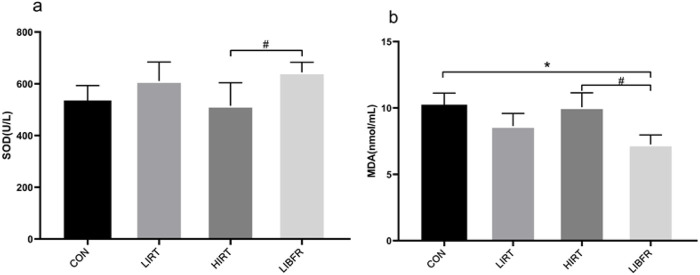
Contents of SOD and MDA in rat serum, **(A)** represents the determination result of SOD, **(B)** represents the determination result of MDA.

As shown in Table 3 and Figure 5, the SOD value of the LIBFR group was the highest, showing a significant difference compared with the HIRT group (P < 0.05). Regarding the MDA index, the MDA concentration value of the LIBFR group was the lowest, showing a significant difference compared with the CON group and the HIRT group (P < 0.05). Compared with the CON group, the MDA concentration in the LIRT group showed a decreasing trend, but no significant difference was observed (P > 0.05).

**TABLE 3:** Contents of SOD and MDA in rat serum.

Groups	CON	LIRT	HIRT	LIBFR	Cohen’s d	p-value
SOD (U/L)	535.26 ± 57.75	610.72 ± 73.34	515.14 ± 89.03	644.23 ± 39.3^#^	0.96	0.136
MDA (nmol/mL)	10.25 ± 0.88	8.64 ± 0.95	10.06 ± 1.08	7.25 ± 0.72*^#^	1.62	0.013

Compared with the CON, group,*P < 0.05 Compared with the HIRT, group, ^#^P < 0.05.

Cohen’s d denotes effect size and p denotes test of statistical significance.

### Nrf2 and Keap1

**FIGURE 6:**
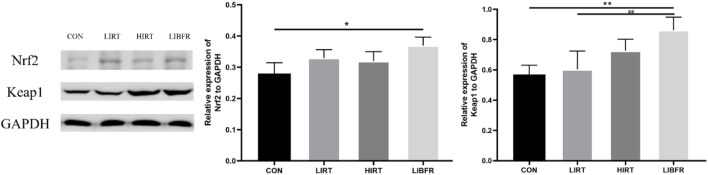
Protein expression of Nrf2 and Keap1 in rat myocardium Compared with the CON group,*P < 0.05,**P < 0.01 Compared with the LIRT group, ^##^P < 0.01.

As shown in Figure 6, compared with the CON group, the expression level of Nrf2 in the myocardium of rats in the LIBFR group was significantly increased (P < 0.05), and the expression level of Keap1 was increased (P < 0.01); compared with the LIRT group, the expression level of Nrf2 in the HIRT group slightly decreased (P > 0.05), and the expression level of Keap1 was increased (P > 0.05); compared with the HIRT group, the expression levels of Nrf2 and Keap1 in the myocardium of rats in the LIBFR group were increased, but no significant difference was observed. Nrf2 metric Cohen’s d = 1.327, p = 0.036, Keap1 metric Cohen’s d = 1.597, p = 0.014.

### Bcl-2 and Bax

**FIGURE 7:**
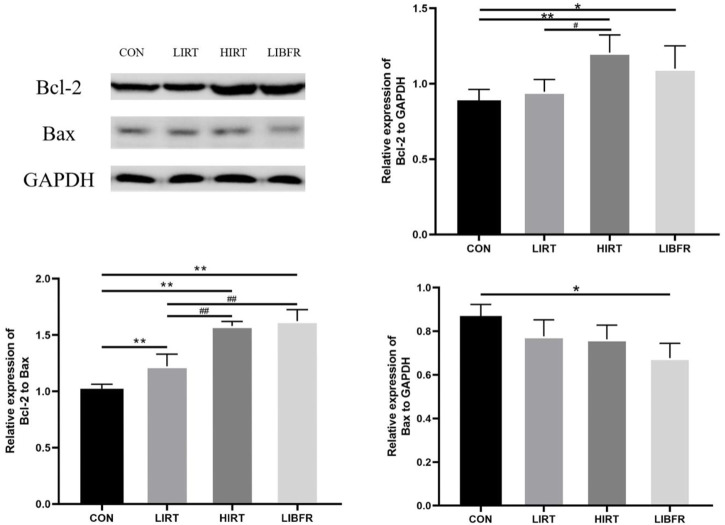
Protein expression of Bcl-2 and Bax in rat myocardium Compared with the CON group,*P < 0.05,**P < 0.01 Compared with the LIRT group, ^#^P < 0.05, ^##^P < 0.01.

As shown in Figure 7, compared with the CON group, the expression level of Bcl-2 in the myocardium of rats in the HIRT group was significantly increased (P < 0.01). Similarly, the expression level of Bcl-2 in the myocardium of rats in the LIBFR group was significantly higher (P < 0.05). On the other hand, the expression level of Bax in the LIBFR group was significantly decreased (P < 0.05). Regarding the Bcl-2/Bax index, the LIRT group, the HIRT group, and the LIBFR group all showed significant increases compared with the CON group (P < 0.01). Additionally, the values of the HIRT and LIBFR groups were significantly higher than those of the LIRT group (P < 0.01). Bcl-2 metric Cohen’s d = 1.60, p = 0.014, Bax metric Cohen’s d = 1.27, p = 0.044.

## Discussion

cTn1 is an important marker for judging myocardial injury in the body, with high specificity and sensitivity. Its content is positively correlated with the degree of myocardial injury and the area of myocardial injury, and is not affected by age, gender, and the location of myocardial injury. (Westermann et al., 2017). After 8 weeks of resistance training, the results showed that the content of cTn1 in the HIRT group was the highest. Combined with the results of CK-MB and HE staining, we infer that excessive training changes the permeability of the myocardial cell membrane, resulting in an increase in the amount of cTn1 entering the blood, which may cause local damage to the myocardial fibers in the HIRT group of rats (Bishop et al., 2019; Mousavi et al., 2009). Some people also think that the abnormal increase in serum cTn1 after high-intensity exercise is due to other factors causing “reversible damage” to the myocardium (Yuli and Songtao, 2020). Therefore, the reasons for the increase in serum cTn1 levels after exercise and its physiological and pathological significance remain to be further studied. By analyzing the LIRT group, the results are showing that although the serum cTnI concentration showed an upward trend compared with the CON group However, for patients with existing, there was no significant difference. Combined with the results of CK-MB, it is suggested that appropriate low-intensity resistance training can have a positive impact on the myocardium and reduce the risk of myocardial infarction. Although there was no significant difference between LIBFR and LIRT, there was a downward trend. The possible reason is that LIBFR regulates the process of cortical excitation and inhibition by improving endothelial function and neural reflex mechanisms (Ma, 2021), thereby normalizing the function of the subcortical vasomotor center, thereby improving the neural and vascular systems. The tourniquet of the limbs leads to a decrease in peripheral vascular resistance and a reduction in the afterload of the heart, thereby achieving the effects of lowering blood pressure, enhancing myocardial contractility, and improving cardiac function. It suggests that low-intensity pressure resistance training can promote myocardial protection and reduce the risk of cardiovascular diseases. However, for patients with existing cardiovascular diseases, the use of blood flow restriction training to explore its underlying mechanisms requires further investigation.

The myocardial antioxidant capacity is regarded as one of the fundamental factors for exercise-induced myocardial protection. Besides the classical antioxidant enzyme SOD, which is commonly used to assess the antioxidant level in the body, its upstream regulatory molecule Nrf2 plays a key role. Nrf2 regulates the expression of various downstream antioxidant enzymes by activating the antioxidant response. This mechanism reflects the overall antioxidant status of the body. Additionally, highly reproducible experimental results have been obtained to support this finding (Chen and Maltagliati, 2018). The results showed that after 8 weeks of training, the antioxidant capacity of rats in each group was evaluated. In both the LIRT and LIBFR groups, SOD activity increased. Additionally, the MDA concentration decreased in both groups. The decrease in MDA concentration was more pronounced in the LIBFR group. Although the LIBFR and LIRT groups trained at the same intensity, the LIBFR group outperformed the LIRT group in terms of antioxidant outcomes (e.g., SOD and MDA), which may be attributed to the incorporation of blood flow restriction into the low-intensity training of the LIBFR group, a factor that increases the cellular response to oxidative stress by decreasing the supply of oxygen, forcing the muscles to work in a hypoxic state, which may lead to the activation of the antioxidant system, thus increasing SOD activity and decreasing MDA production (Song et al., 2023). The Nrf2 in the LIRT group and the LIBFR group both increased, and the protein expression level of Keap1 was the highest in the LIBFR group. This indicates that moderate and low-intensity exercise is beneficial to the improvement of the body’s antioxidant capacity, causes less damage to the myocardial cell membrane, delays myocardial aging, and can exert a certain protective effect on the myocardium. Moreover, low-intensity resistance combined with blood flow restriction training has a more obvious improvement effect in this aspect compared to simple low-intensity resistance training. The SOD activity of rats in the HIRT group was the lowest, the MDA concentration was higher than that in the LIBFR and LIRT groups, and the Nrf2 value was lower than that in the LIRT and LIBFR groups. In terms of Keap1 protein expression, the HIRT group was higher than the CON group and the LIRT group but lower than the LIBFR group. This suggests that long-term high-intensity exercise may not be conducive to the development of the body’s antioxidant capacity and causes greater damage to the myocardial cell membrane. Moreover, after a long period of high-load exercise, the activity of antioxidant enzymes decreases and the generation of free radicals increases. This is immediately increasing the activity of antioxidant enzymes after exercise is not sufficient to eliminate the increased free radicals, and the accumulation of free radicals in the body causes lipid peroxidation of the cell membrane, resulting in myocardial injury (Lu et al., 2015). However, in this experiment, the myocardial MDA content of rats in the HIRT group after exercise was almost the same as that of CON rats, with no significant difference. We speculate that the reason for this is that the MDA concentration was relatively high immediately after exercise, and gradually decreased with the extension of the recovery time after exercise (Zhao et al., 2013), reaching the lowest point at 24 h. This indicates that although a large number of free radicals are generated in the myocardial tissue, no severe lipid peroxidation occurs. This suggests that there is a relatively powerful anti-free radical system within the myocardium, which helps to maintain myocardial function. It may also suggest that the heart, as the center of life, possesses an exceptionally strong ability to maintain internal environment homeostasis. Understanding the underlying mechanisms of this process requires continuous exploration.

Cell apoptosis can eliminate functionally damaged and abnormal cells in organisms and plays a very important regulatory role in maintaining the stability of the internal environment of organisms. Reactive oxygen species, cytokines, etc., Can all induce the occurrence of cell apoptosis. Bcl-2 and Bax play important functions in the apoptotic process. The two competitively form dimers with each other and can achieve dual regulation of apoptosis through the influence on mitochondrial structure. Among them, the Bcl-2 gene has the function of inhibiting apoptosis, and the Bax gene has the function of enhancing apoptosis (Warren et al., 2019). In this experiment, we detected the ratios of Bcl-2, Bax, and Bcl-2/Bax. Compared with the CON group, the expression levels of the anti-apoptotic related protein Bcl-2 increased, the expression level of Bax decreased, and the ratio of Bcl-2/Bax increased in the LIRT group, the HIRT group, and the LIBFR group. The expression of Bcl-2 protein in the LIRT group was lower than that in the HIRT group and the LIBFR group. The HIRT group had the highest expression of Bcl-2 protein, but the expression of Bax protein was lower than that in the LIBFR group, which made the final Bcl-2/Bax value slightly lower than that in the LIBFR group. It indicates that resistance training can increase the activity of Bcl-2, increase its affinity with Bax, prevent Bax from dissociating from the complex, destroy the integrity of the mitochondrial membrane, and exert its pro-apoptotic effect (Siddiqui et al., 2015). Low-intensity resistance training could inhibit cell apoptosis, and blood flow restriction combined with low-intensity resistance training could better inhibit myocardial cell apoptosis. We collected a large number of relevant studies on blood flow restriction and did not find the mechanism by which this method affects the expression of myocardial anti-apoptotic related proteins. We speculate that it may be because the tourniquet on the limb causes compression of blood vessels and a decrease in the amount of blood returning to the heart. To maintain normal blood supply to the body and stable blood pressure, the heart needs to make appropriate adjustments, such as increasing the heart rate to maintain the cardiac output at the same level, or enhancing myocardial contractility to ensure adequate cardiac blood supply. After long-term ischemic training, the heart has undergone positive adaptive changes, resulting in a higher anti-apoptotic level compared to the control group.

Although this study used a rat model to assess the effects of LIBFR on cardiovascular health, these findings have potential clinical applications, especially for patients with cardiovascular disease. Although rat models provide valuable data on physiological and pathological mechanisms, there are some differences between rats and humans in terms of cardiovascular responses, metabolic rates and oxidative stress responses. However, the positive effects of LIBFR and HIRT training on cardiovascular fitness may provide an effective form of exercise for people with cardiovascular disease. LIBFR training may have a positive impact on people with cardiovascular disease by enhancing antioxidant responses, improving haemodynamics and promoting microvascular function. Whilst HIRT also has benefits in terms of improving endurance and cardiovascular fitness, it may not be suitable for all patients, particularly those with more advanced disease, due to its greater metabolic load. Therefore, LIBFR offers a milder and more suitable training modality for patients with cardiovascular disease that can enhance blood oxygen supply and vascular health while avoiding overload. Nevertheless, translating these experimental results to human populations, particularly those with cardiovascular disease, requires further clinical studies. In particular, the long-term efficacy and safety of LIBFR and HIRT in patients with different training intensities, frequencies and durations need to be evaluated.

## Research limitations and perspectives

Limitations of this study mainly include the small sample size, the use of only animal models, and the lack of direct exploration of the specific molecular mechanisms of LIBFR. In addition, the short observation period of the study did not allow for assessment of the long-term effects of LIBFR. Despite our focus on the myocardial effects of LIBFR, future studies should be conducted over longer time spans and in broader populations, especially for clinical validation in patients with cardiovascular disease and in the elderly population. Meanwhile, studies need to further explore the mechanism of action of LIBFR at the molecular level to deepen the understanding of its antioxidant and anti-apoptotic functions.

In terms of clinical relevance, LIBFR, as a low-intensity, high-efficiency training modality, may provide a safe and effective alternative for individuals who are unable to perform high-intensity exercise, especially patients with cardiovascular disease and the elderly. Future research should focus on clinical trials to validate the actual impact of LIBFR on cardiac health in these high-risk groups and to explore its long-term effects and safety of application.

The motivation for this study was to explore an exercise modality suitable for cardiovascular patients and older adults to provide cardioprotection through LIBFR training. In contrast to the existing literature, our study fills the research gap on LIBFR in terms of cardiac function, especially its potential effects on myocardial damage in the context of low-intensity training combined with blood flow restriction, providing a different perspective from existing heart health studies that focus on aerobic or high-intensity training.

## Conclusion

Low-intensity blood flow restriction training can effectively reduce cTn1 in rat serum, decrease cardiomyocyte apoptosis, and improve antioxidant capacity, which has a certain protective effect on the myocardium. In addition, we observed that the MVCC values of the LIBFR group were comparable to those of the HIRT group, suggesting that LIBFR may have similar effects in muscle activation as high-intensity training.

## Data Availability

The datasets presented in this article are not readily available due to confidentiality restrictions. Requests to access the anonymized datasets should be directed to shangguan0202@gmail.com.
